# Based Interprofessional Didactic for Emergency Management of Snakebites

**DOI:** 10.5070/M5.61957

**Published:** 2026-10-31

**Authors:** Nicky Chung, Anjali Cherukuri, Debayan Guha, Joshua Silverberg, Oark Ahmed, Ibrahim Farooqui, Jacob Greenspan, Anthony Scoccimarro, Jeremy Sperling, Ariella Gartenberg, Maninder Singh

**Affiliations:** * NYC Health + Hospitals/Jacobi, Department of Emergency Medicine, Bronx, NY

## Abstract

**Audience:**

This simulation is intended for Post-Graduate Level 1–4 Residents and Attending Physicians.

**Introduction:**

Although uncommon in the Northeastern United States, emergency physicians may encounter exotic envenomation through zoological institutions, research facilities, or privately owned exotic reptiles.^1^ These cases present unique diagnostic and logistical challenges because management frequently requires species-specific antivenom and early consultation with regional toxicology experts.^1^,^2^,^3^ Emergency providers may be unfamiliar with such presentation, evaluation, and treatment steps. Simulation-based education can bridge this gap by allowing interprofessional teams, including residents, attendings, nurses, and pharmacists, to practice coordinated management in a safe environment.^4^,^5^ This project evaluates the feasibility, learner-reported preparedness, and educational outcomes associated with a multidisciplinary snakebite simulation.

**Educational Objectives:**

By the end of the simulation, participants will be able to recognize and appropriately manage neurotoxic snakebite envenomation, including initiating first-line management and toxicology consultation, coordinating antivenom administration, recognizing and treating antivenom-associated anaphylaxis, assessing and managing progressive respiratory failure, demonstrating effective interprofessional communication using TeamSTEPPS principles,^4^ and coordinating timely consultation, handoff, and disposition with toxicology, pharmacy, and the critical care team.

**Educational Methods:**

High-fidelity simulation will be used to provide an immersive environment. TeamSTEPPS (team strategies and tools to enhance performance and patient safety), an evidence-based communication training toolkit, will be used.^4^ High-fidelity simulation for snakebite education has been shown to enhance clinical skills, foster teamwork, and enable more timely and proficient provider care in snakebite cases.^5^ The simulation will depict an Indian Cobra snakebite case with evolving neurotoxicity and potential anaphylaxis after antivenom. Teams will manage airway, antivenom reconstitution, and anaphylaxis using TeamSTEPPS principles.^4^ Scenario branches depend on learner actions; facilitators will provide physiologic cues accordingly.

**Research Methods:**

The study consisted of a prospective, single-center, pre/post simulation-based educational pilot study. Physician participants completed pre- and post-surveys and objective multiple-choice tests. Observers used checklists to assess performance. Given the pilot nature of the simulation, no *a priori* sample size calculation was performed. The simulation was conducted using an interprofessional team model that included emergency medicine physicians, bedside nurses, pharmacists, and a toxicologist. Although all disciplines participated in the simulation to replicate real-word emergency department management of snakebite envenomation, only physician participants completed the pre- and post-intervention knowledge and confidence assessments. The educational study was designed to evaluate physician learning outcomes.

**Results:**

Seven emergency medicine physicians from varying training levels participated in the simulation, with a post-test response rate of 85.7% (n=6). Participants demonstrated significant improvements in self-reported knowledge regarding overall snakebite management (*p* = 0.042), appropriate first aid (*p* = 0.010), antivenom complications (*p* = 0.010), and recognition of both neurotoxic *(p* = 0.012) and hemotoxic envenomation patterns (*p* = 0.010). Confidence in administering antivenom (*p* = 0.025) and working effectively within a team during high-stress emergencies (*p* = 0.042) improved as well. No significant improvement was observed in objective multiple-choice knowledge scores. Critical action checklists demonstrated that all members were able to identify key clinical findings, communicate with toxicology, and recognize anaphylaxis, although adherence to antivenom preparation steps varied.

**Discussion:**

The simulation-based didactic was feasible and associated with improvements in self-reported knowledge, confidence, and perceived teamwork related to snakebite management among physician participants. However, objective knowledge scores did not significantly improve, highlighting the need for continued reinforcement of cognitive knowledge and anti-venom related procedural and systems-based skills. Given the pilot design and limited sample size, these findings should be interpreted as preliminary and hypothesis-generating. Future iterations of the simulation will include larger sample sizes, expand emphasis on antivenom logistics, and incorporate deliberate practice stations to ensure both cognitive and procedural fidelity.

**Topics:**

Snakebite, antivenom, anaphylaxis, neurotoxicity, communication, teamwork.

## USER GUIDE

**Table:** 

List of Resources:	
Abstract	46
User Guide	48
Instructor Materials	52
Operator Materials	59
Debriefing and Evaluation Pearls	62
Simulation Assessment	66
Appendix A: Critical Action Checklist	72

**Learner Audience:**

Post-Graduate Level 1–4 Residents and Attending Physicians

**Time Required for Implementation:**

**Preparation:** 30 minutes

**Time for case:** 15 minutes

**Time for debriefing:** 20 minutes

**Recommended Number of Learners per Instructor:**

6

**Topics:**

Snakebite, antivenom, anaphylaxis, neurotoxicity, communication, teamwork.

**Objectives:**

By the end of the simulation scenario, participants will be able to:

1. Verbally identify clinical signs of neurotoxic envenomation (eg, ptosis, bulbar weakness, respiratory compromise) and initiate appropriate first-line management, including limb immobilization and early toxicology consultation, as demonstrated on the simulation critical action checklist.
2. Demonstrate effective coordination of antivenom administration, including identification of the need for antivenom, consultation with appropriate resources, communication with pharmacy or nursing teams, and safe initiation of therapy using institutional protocols, with completion of all required procedural steps as assessed by the simulation checklist.
3. Recognize early signs of antivenom-induced anaphylaxis and administer appropriate interventions (intramuscular epinephrine, airway support, adjunctive therapies), as measured by timely completion of critical actions during the simulation.
4. Demonstrate appropriate assessment and management of progressive respiratory failure due to neurotoxin effects, including recognition of indications for endotracheal intubation, as evaluated by facilitator observation.
5. Demonstrate effective interprofessional communication using TeamSTEPPS principles (call-outs, check-backs, closed-loop communication, role clarity), as assessed using the teamwork evaluation checklist.^4^
6. Coordinate timely consultation and handoff with toxicology, pharmacy, and the intensive care unit (ICU) team, as demonstrated by appropriate communication, disposition planning, and documentation.

### Linked objectives and methods

The goals and learning objectives of this curriculum are best achieved through a high-fidelity, interprofessional simulation format. This format allows participants to practice recognition and management of a rare but high-stakes emergency - neurotoxic snakebite envenomation - in a realistic and controlled environment. Although venomous snakebites encountered in the Northeastern United States most commonly involve native pit vipers, the authors intentionally selected an Indian cobra (*Naja naja*) scenario for this curriculum. The case was designed to emphasize rare but high-acuity emergency medicine competencies, including recognition of progressive neurotoxic envenomation, early airway management, species-specific antivenom selection, and management of antivenom-associated anaphylaxis, that are difficult to teach using more common regional species such as copperheads [objective 1]. In addition, emergency physicians practicing in urban centers may encounter exotic snake envenomation through zoos, private collectors, research facilities, or illegal exotic pet ownership. Although uncommon, these cases require rapid consultation with regional poison centers and toxicology experts because species-specific antivenom and treatment pathways differ substantially from those used for North American pit vipers.

Since most providers in the Northeastern United States have limited real-world exposure to venomous snakebites, simulation offers a safe, reproducible setting to develop clinical reasoning, procedural skills, teamwork behaviors, and systems-based workflows that cannot be effectively taught through lecture alone. The authors selected a format that aligns with our objectives targeting knowledge acquisition, antivenom preparation, anaphylaxis management, and team communication. This format provides observable, measurable behaviors that can be evaluated using standardized critical action checklists, allowing simultaneous assessment across multiple learning domains. The cognitive domain (knowledge) is assessed through learners’ ability to recognize neurotoxic envenomation, interpret the patient’s evolving clinical presentation, and make appropriate diagnostic and therapeutic decisions (objectives 2 and 3). The psychomotor domain (procedural) is evaluated by procedural tasks, such as performing the primary assessment, preparing and administering antivenom, recognizing and treating anaphylaxis, and initiating airway management when indicated (objective 4). The affective domain (team performance) is assessed through interprofessional behaviors including leadership, role delegation, closed-loop communication, situational awareness, and collaboration with nursing, pharmacy, and toxicology consultants (objectives 5 and 6). Simulation also allows learners to experience progressive physiological deterioration, respond to time-sensitive clinical decisions, and coordinate multidisciplinary care in a manner that closely reflects real-world emergency department management of snakebite envenomation.^5^

This curriculum was developed using principles from Kolb’s Experiential Learning Theory, which integrates experience, theory and simulation.^6^ The scenario design specifically follows the PEARLS blended debriefing model, integrating directive feedback, focused facilitation, and self-reflection to promote deeper understanding and retention.^7^ Team communication elements align with the TeamSTEPPS framework, ensuring that interprofessional collaboration, role clarity, and closed-loop communication are explicitly practiced and assessed.^4^ Furthermore, this format directly aligns with the SMART learning objectives, which are achievable, measurable and observable within the single simulation session.

### Recommended pre-reading for instructor

- Goldfrank LR, Hoffman RS, Howland MA, Lewin NA, Nelson LS, Flomenbaum NE. Antivenom (crotaline and elapid). In: Nelson LS, Howland MA, Lewin NA, Goldfrank LR, Hoffman RS, Flomenbaum NE, eds. *Goldfrank's Toxicologic Emergencies*. 10th ed. McGraw-Hill Education; 2015:1537–1556.

### Learner responsible content

- Walls RM, Hockberger RS, Gausche-Hill M, eds. *Rosen's Emergency Medicine: Concepts and Clinical Practice*. 10th ed. Elsevier; 2023:690–708.e3.
- Learners were provided optional preparatory reading from Rosen’s emergency medicine; completion of assigned reading was not tracked.

### Results and tips for successful implementation

The data was collected utilizing web-based survey assessment. Pre- and post-simulation surveys, as well as critical action checklists completed during the simulation, were collected for all participants. Pre- and post-simulation surveys utilized a 5- point Likert scale for subjective assessment and multiple-choice questions for objective knowledge assessment. Surveys were anonymous and did not contain any personal health information. Data was stored securely in Excel and analyzed anonymously. Given the pilot educational design, analyses focused primarily on descriptive statistics and exploratory comparisons of pre- and post-simulation outcomes. Likert-scale responses were analyzed as approximately interval-level data. Pre- and post-simulation responses were compared using paired t-tests. The distribution of paired differences was assessed graphically and demonstrated no substantial deviation from normality or significant outliers. Statistical significance was defined as *p*<0.05. Due to the limited sample size and multiple comparisons performed, findings were interpreted cautiously. Descriptive statistics were used to summarize participant characteristics, learner perceptions, and checklist performance.

Seven learners participated in the simulation, with a post-test response rate of 85.7% (n=6). Participants were from various training levels including: PGY 1 (28.6%, n=2), PGY 2 (14.3%, n=1), PGY 3 (28.6%, n=2), PGY 4 (14.3%, n=1), and attending physicians (14.3%, n=1). In terms of subjective assessments pre- and post-simulation, there was improvement in self-reported:

- ED provider knowledge regarding how to manage patients presenting with a snakebite (Likert mean 3.17 SD 0.41 versus 4.0 standard deviation (SD) 0.63, p = 0.042)
- ED provider knowledge regarding how to provide first aid for a snakebite (Likert mean 3.00 SD 0.63 versus 4.33 SD 0.82, *p* = 0.010)
- ED provider knowledge regarding the potential complications that can arise from antivenom (Likert mean 2.83 SD 0.41 versus 4.17 SD 0.75, *p* = 0.010)
- ED provider familiarity with the clinical signs of envenomation from neurotoxic snakes (Likert mean 3.0 SD 0.00 versus 4.0 SD 0.63, *p* = 0.012)
- ED provider familiarity with the clinical signs of envenomation from hemotoxic snakes (Likert mean 2.67 SD 0.52 versus 4.0 SD 0.63, *p* = 0.010)
- ED provider confidence in effectively working as part of a team in a high-stress emergency situation (Likert mean 3.17 SD 0.75 versus 4.0 SD 0.63, *p* = 0.042)
- ED provider confidence administering antivenom in the setting of a snakebite (Likert mean 3.17 SD 0.75 versus 3.83 SD 0.75, *p* = 0.025)

Pre- and post-simulation demonstrated no significant difference in terms of self-reported:

- ED provider knowledge regarding how to obtain antivenom (Likert mean 3.17 SD 0.75 versus 4.0 SD 0.89, *p* = 0.093)
- ED provider confidence in assessing the severity of a snakebite (Likert mean 3.0 SD 0.89 versus 3.67 SD 0.52, *p* = 0.175)
- ED provider confidence in providing first aid to a patient with a snakebite (Likert mean 3.17 SD 0.75 versus 4.0 SD 0.63, *p* = 0.093)
- Objective multiple-choice questionnaire pre- and post-simulation (52.3% versus 52.4%, *p* = 0.60)

Participants felt that the simulation scenario was realistic (average Likert scale of 4.33 SD 0.82), helped them practice real-world skills for managing a snakebite in the ED (average 4.17 SD 0.75), and prepared them to manage future patients with snakebites and related complication (average 4.17 SD 0.75). Participants also felt that the debriefing session was helpful in consolidating what they learned from the simulation (average 4.33 SD 0.82).

Overall, this pilot study suggests that the interprofessional simulation was feasible and associated with improvement in self-reported knowledge, confidence, and teamwork behaviors, while also identifying persistent gaps in objective knowledge and antivenom-related logistics. Learners also rated the simulation as realistic and beneficial for practicing emergency management skills. Given the pilot design and limited sample size, these findings should be interpreted as preliminary and hypothesis-generating.

Implementation of this scenario highlighted several important lessons. First, although learners improved in perceived knowledge, conceptual understanding, and crisis resource management skills, objective knowledge and procedural skills related to antivenom preparation and acquisition remained unchanged. This finding highlights an important distinction between perceived preparedness and measurable knowledge acquisition. The absence of improvement on objective testing may reflect several factors, including the limited sample size, the difficulty of detecting changes in cognitive outcomes after a single simulation exposure, and the possibility that simulation-based education preferentially enhances clinical reasoning, procedural readiness, and teamwork behaviors rather than isolated factual recall. Additionally, the objective assessment focused primarily on knowledge-based questions and may not have fully captured the procedural and team-based competencies emphasized by the simulation. Future iterations may incorporate additional preparatory materials, spaced educational interventions, and repeated simulation exposure to reinforce cognitive knowledge retention.

Findings further revealed a clear systems-learning gap: many trainees were unfamiliar with institutional workflows, emphasizing the need to incorporate operational logistics, such as where antivenom is stored and how it is obtained, directly into the simulation and debrief. These findings should be interpreted cautiously given the pilot nature of this study and the limited sample size. Only seven participants were enrolled, with six completing the post-simulation assessment, limiting statistical power and generalizability of findings. Consequently, the pilot should be viewed as demonstrating preliminary feasibility and educational promise rather than definitive effectiveness. In addition, completion of optional preparatory reading was not assessed, limiting evaluation of traditional asynchronous learning contribution to baseline knowledge. Future iterations of the simulation will include larger sample sizes, expand emphasis on antivenom logistics, and incorporate deliberate practice stations to ensure both cognitive and procedural fidelity.

### Associated content

Critical Actions Checklist

## Figures and Tables

**Table:** 

**#1**	**Picture of snakebite wound**

**Table:** 

**Vitals:**	Heart Rate (HR) 115	Blood Pressure (BP) 115/80
	Respiratory Rate (RR) 24	Temperature (T) 98.6°F
	Oxygen Saturation (O_2_Sat) 98% on room air (RA)	

**Figure:**
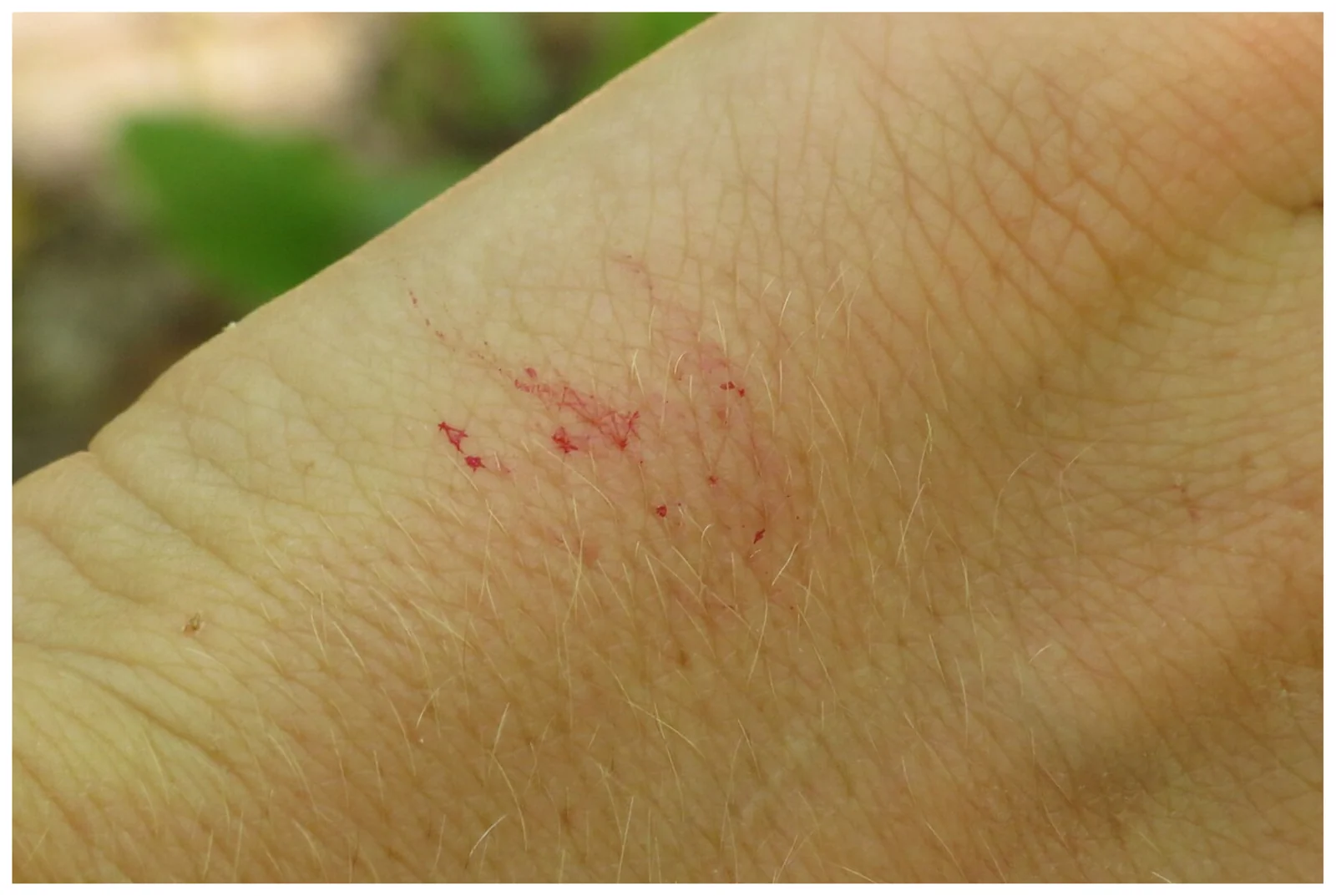


**Table:** SIMULATION EVENTS TABLE:

Minute (state)	Participant action/ trigger	Patient status (simulator response) & operator prompts	Monitor display (vital signs)
0:00 (Baseline)	ABCs AMPLE history Identify neurotoxic envenomation Immobilize affected extremity (if not performed, address in the debriefing) Call toxicology consult. Ask for antivenom	The patient reports a metallic taste in his mouth. The patient has bilateral eyelid drooping. If participants do not call toxicology consult by 4 minutes, the nurse prompts participants: Should we call the toxicology consult? The patient will then remember he has the antivenom and alert the team. If learners attempt to independently prepare antivenom without appropriate resources, the nurse should state: “I have not prepared this medication before. Should we confirm the reconstitution instructions with pharmacy or toxicology?”	T: 98.6° F HR: 115 BP: 115/80 RR: 24 O2: 98% RA
4–6 minutes (If no antivenom given): Appropriate airway management	Learners should intubate the patient. Appropriate airway management: SpO2 improves to 99%, ventilation is adequate, and the case proceeds to ICU disposition	Patient becomes lethargic, taking shallow breaths Nurse: The patient isn’t looking so good. He’s becoming hypoxic. The patient is intubated and oxygenation improves.	HR: 62 BP: 70/30 RR: 8 SpO2: 80% → 99%
4–6 minutes (If no antivenom given): Delayed airway management	Learners should intubate the patient. Delayed airway management: Progressive respiratory muscle paralysis develops. The patient becomes apneic requiring emergent intubation.	Patient becomes lethargic, taking shallow breaths Nurse: “The patient isn’t looking so good. He’s becoming hypoxic."	HR: 62 BP: 70/30 RR: 8 SpO2: 80%
4–6 minutes (If antivenom given)	Learners should recognize anaphylaxis following antivenom administration. Administer IM epinephrine.	Patient develops urticaria, hoarse voice, and lungs have diffuse wheezing bilaterally Patient complaints of itchiness, hives, and voice changing Nurse: the patient’s blood pressure is dropping and he has increased work of breathing	HR: 120 BP: 90/40 RR: 24 SpO2: 95%
6–10 minutes (If no epinephrine given)	Learners should intubate the patient as he is losing his airway. SpO2 improves to 99%, ventilation is adequate, and the case proceeds to ICU disposition.	The patient becomes lethargic. Exam shows oropharyngeal swelling and decreased breath sounds bilaterally.	HR: 120 BP: 70/43 RR: 20 SpO2: 75%
6–10 minutes (If epinephrine given)	Learners should disposition to the ICU. Case concludes	Patient improves	HR: 105 BP: 135/90 RR: 14 SpO2: 98%
(Case Completion)			

**Figure:**
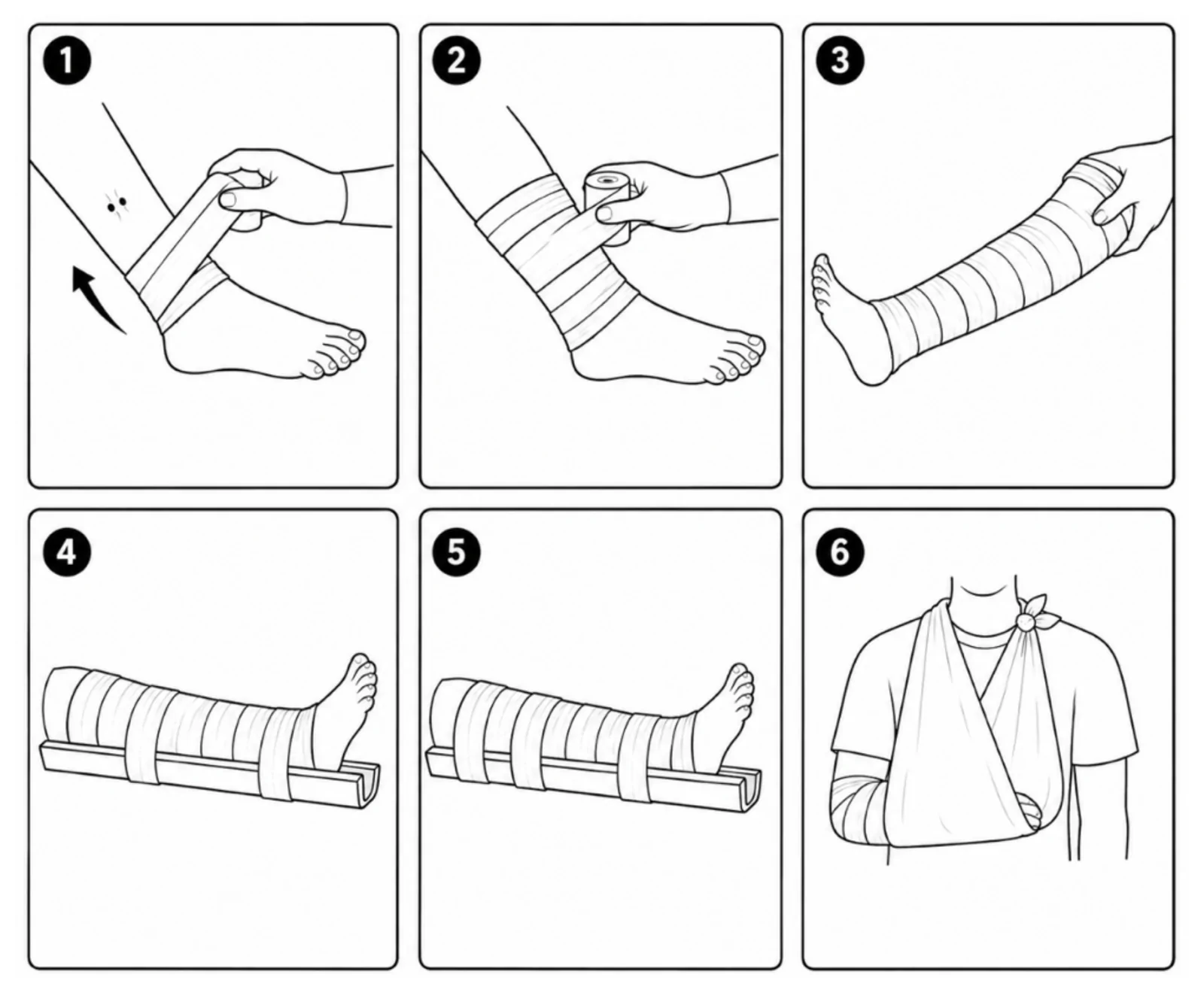


**Table:** 

**Critical Actions:** Verbalizes that the Indian Cobra (*Naja naja*) snake produces neurotoxic envenomation and communicates the implications for patient management (need for airway monitoring and early toxicology consultation) Perform the emergency medicine airway, breathing, circulation (“ABC”): Intubation? Bilateral breath sounds? Equal pulses? Mark the site (if needed)? Immobilize affected extremity? Avoidance of tourniquets or incision/suction? IV access and labs ordered: complete metabolic panel (CMP), complete blood count (CBC), fibrinogen, prothrombin time (PT) and partial thromboplastin time (PTT), creatinine phosphokinase (CPK), venous blood gas (VBG) Ask for antivenom (snake handler brings the antivenom with him from the zoo) Perform Snakebite First Aid: Splint the extremity and elevate to the level of the heart Call the Toxicology consult/Snakebite consult Coordinate antivenom administration with pharmacy/nursing team and confirm appropriate preparation and monitoring requirements Connect the patient to the monitor for antivenom administration Monitor for anaphylaxis. Pretreatment is generally not needed. Verbally recognize the development of anaphylaxis (identifies hypotension, urticaria, wheezing, or airway involvement) and promptly administers intramuscular epinephrine Disposition: The patient should be admitted to an appropriate ICU setting for monitoring and management. Specific admitting services and ICU location may vary by institution.	0:00

**Table:** 

	Milestone	Did not achieve level 1	Level 1	Level 2	Level 3
1	**Emergency Stabilization (PC1)**	□ Did not achieve Level 1	□ Recognizes abnormal vital signs	□ Recognizes an unstable patient, requiring intervention Performs primary assessment Discerns data to formulate a diagnostic impression/plan	□ Manages and prioritizes critical actions in a critically ill patient Reassesses after implementing a stabilizing intervention
2	**Performance of focused history and physical (PC2)**	□ Did not achieve Level 1	□ Performs a reliable, comprehensive history and physical exam	□ Performs and communicates a focused history and physical exam based on chief complaint and urgent issues	□ Prioritizes essential components of history and physical exam given dynamic circumstances
3	**Diagnostic studies (PC3)**	□ Did not achieve Level 1	□ Determines the necessity of diagnostic studies	□ Orders appropriate diagnostic studies. Performs appropriate bedside diagnostic studies/procedures	□ Prioritizes essential testing Interprets results of diagnostic studies Reviews risks, benefits, contraindications, and alternatives to a diagnostic study or procedure
4	**Diagnosis (PC4)**	□ Did not achieve Level 1	□ Considers a list of potential diagnoses	□ Considers an appropriate list of potential diagnosis May or may not make correct diagnosis	□ Makes the appropriate diagnosis Considers other potential diagnoses, avoiding premature closure
5	**Pharmacotherapy (PC5)**	□ Did not achieve Level 1	□ Asks patient for drug allergies	□ Selects an medication for therapeutic intervention, consider potential adverse effects	□ Selects the most appropriate medication and understands mechanism of action, effect, and potential side effects Considers and recognizes drug-drug interactions
6	**Observation and reassessment (PC6)**	□ Did not achieve Level 1	□ Reevaluates patient at least one time during case	□ Reevaluates patient after most therapeutic interventions	□ Consistently evaluates the effectiveness of therapies at appropriate intervals
7	**Disposition (PC7)**	□ Did not achieve Level 1	□ Appropriately selects whether to admit or discharge the patient	□ Appropriately selects whether to admit or discharge Involves the expertise of some of the appropriate specialists	□ Educates the patient appropriately about their disposition Assigns patient to an appropriate level of care (ICU/Tele/Floor) Involves expertise of all appropriate specialists
9	**General Approach to Procedures (PC9)**	□ Did not achieve Level 1	□ Identifies pertinent anatomy and physiology for a procedure Uses appropriate Universal Precautions	□ Obtains informed consent Knows indications, contraindications, anatomic landmarks, equipment, anesthetic and procedural technique, and potential complications for common ED procedures	□ Determines a back-up strategy if initial attempts are unsuccessful Correctly interprets results of diagnostic procedure
20	**Professional Values (PROF1)**	□ Did not achieve Level 1	□ Demonstrates caring, honest behavior	□ Exhibits compassion, respect, sensitivity and responsiveness	□ Develops alternative care plans when patients’ personal beliefs and decisions preclude standard care
22	**Patient centered communication (ICS1)**	□ Did not achieve level 1	□ Establishes rapport and demonstrates empathy to patient (and family) Listens effectively	□ Elicits patient’s reason for seeking health care	□ Manages patient expectations in a manner that minimizes potential for stress, conflict, and misunderstanding. Effectively communicates with vulnerable populations, (at risk patients and families)
23	**Team management (ICS2)**	□ Did not achieve level 1	□ Recognizes other members of the patient care team during case (nurse, techs)	□ Communicates pertinent information to other healthcare colleagues	□ Communicates a clear, succinct, and appropriate handoff with specialists and other colleagues Communicates effectively with ancillary staff

## INSTRUCTOR MATERIALS

**Case Title:** Snake Bite with Antivenom-Associated Anaphylaxis

**Case Description & Diagnosis (short synopsis):** A patient was bitten on the hand by an Indian Cobra snake at the Bronx Zoo and subsequently brought to your emergency department. He was brought with anti-venom from the zoo’s snake handler, who did not disclose this initially to the team because it was forgotten in the chaos of the transfer. Because this is a neurotoxic snake, antivenom is the definitive management. Expert consultation is key in determining the correct antivenom for treatment of the snakebite. Toxicology serves as a consultation resource rather than directing all clinical decisions. Learners are expected to recognize neurotoxic envenomation, initiate stabilization, obtain appropriate diagnostics, identify the need for antivenom, and coordinate multidisciplinary management.

If antivenom is not given in this scenario, the patient will require intubation for respiratory paralysis. If antivenom is administered, the scenario introduces a potential hypersensitivity reaction requiring recognition and management of anaphylaxis. This branch is designed to assess preparedness for antivenom-associated complications, rather than represent an expected outcome of therapy. Furthermore, this scenario intentionally uses an exotic species to simulate a low-frequency, high-acuity emergency requiring rapid toxicology consultation, species-specific antivenom acquisition, advanced airway management, and coordinated interprofessional care.

**Equipment or Props Needed:**

Cardiac monitor

Splinting supplies

- Orthoglass or plaster
- Soft roll
- Stockinette
- Ace wrap

IV supplies

- 18-gauge angiocatheter
- IV tubing

Medications

- Antivenom (stored in a cooler)
- Epinephrine

Airway equipment

- Nasal cannula
- Non-rebreather mask
- Bag valve mask
- Laryngoscope
- Endotracheal tube and stylet

**Embedded participants needed:**

Patient

Nurse

Herpetologist (Optional)

Toxicologist

**Stimulus Inventory:**

**Background and brief information:** The patient is brought to a large urban academic ED by ambulance from the Bronx Zoo. He presents with an Indian Cobra snakebite to the right hand. His vitals are noted at: HR 115 bpm, RR 24, BP 115/80, SpO2 98% on RA, Temp 98.6° F. Physical exam demonstrates a bite wound to the right hand with mild surrounding erythema. He has drooping of the eyelids and a metallic taste in the mouth. He is tachycardic with regular rhythm and clear breath sounds.

**Initial presentation:** The patient is hemodynamically stable upon arrival. He arrives with a cooler of antivenom with him for the snake (but the team needs to ask for it to get it).

**How the scenario unfolds:** The team should call for a Toxicology consult because most antivenoms are now manufactured in a lyophilized (freeze-dried) powder for long-term stability and storage. The vial is then gently swirled to ensure the powder is fully dissolved and the antivenom is reconstituted for intravenous administration. **If the team does not administer antivenom**, the neurologic symptoms progress. The patient starts taking shallower breaths and becomes more tachycardic. The patient starts to slur his speech. If antivenom is not given, the symptoms progress to respiratory arrest requiring intubation. **If the team gives antivenom**, providers will need to place the patient on a monitor. The patient develops signs of anaphylaxis shortly after antivenom administration. This includes an urticarial pruritic rash, hoarse voice, and wheezing. **If the team does not give epinephrine**, the patient will lose their airway, prompting the team to intubate. **If the team gives epinephrine**, the patient improves. The patient will require a critical care team consultation for ICU-level monitoring. The admitting service and ICU location will depend on institutional resources, local toxicology recommendations, and the nature of the envenomation. This concludes the case.

**Critical actions:**

1. Verbalizes that the Indian Cobra (*Naja naja*) snake produces neurotoxic envenomation and communicates the implications for patient management (need for airway monitoring and early toxicology consultation)
2. Perform the emergency medicine airway, breathing, circulation (“ABC”):
  - Intubation?
  - Bilateral breath sounds?
  - Equal pulses? Mark the site (if needed)? Immobilize affected extremity? Avoidance of tourniquets or incision/suction?
3. IV access and labs ordered: complete metabolic panel (CMP), complete blood count (CBC), fibrinogen, prothrombin time (PT) and partial thromboplastin time (PTT), creatinine phosphokinase (CPK), venous blood gas (VBG)
4. Ask for antivenom (snake handler brings the antivenom with him from the zoo)
5. Perform Snakebite First Aid: Splint the extremity and elevate to the level of the heart
6. Call the Toxicology consult/Snakebite consult
7. Coordinate antivenom administration with pharmacy/nursing team and confirm appropriate preparation and monitoring requirements
8. Connect the patient to the monitor for antivenom administration
9. Monitor for anaphylaxis. Pretreatment is generally not needed.
10. Verbally recognize the development of anaphylaxis (identifies hypotension, urticaria, wheezing, or airway involvement) and promptly administers intramuscular epinephrine
11. Disposition: The patient should be admitted to an appropriate ICU setting for monitoring and management. Specific admitting services and ICU location may vary by institution.

**Case Title:** Snake Bite with Antivenom-Associated Anaphylaxis

**Chief Complaint:** Snakebite from an Indian Cobra snake to the right hand at the Bronx Zoo.

**General Appearance:** Awake and alert

**Primary Survey:**

- **Airway:** Intact
- **Breathing:** Lungs clear to auscultation
- **Circulation:** Tachycardic, Regular. BP within normal limits

**History:**

- **History of present illness: A** 21-year-old male zoo employee presents to the emergency department with right hand pain and a metallic taste in his mouth after being bitten on the right hand by an Indian cobra (*Naja naja)* while cleaning an enclosure at the Bronx Zoo. The bite occurred approximately 30 minutes prior to arrival. Immediately after the bite, the patient notified coworkers, who immobilized the affected extremity and transported him by EMS. The patient reports progressive hand pain and a metallic taste in his mouth. He denies loss of consciousness or other injuries. His tetanus vaccination is up-to-date.
- **Past medical history:** none
- **Past surgical history:** none
- **Patient’s medications:** none
- **Allergies:** none reported
- **Social history:** No daily alcohol use or recreational use of illicit substances.
- **Family history:** none

**Secondary Survey/Physical Examination:**

- **General appearance:** Awake, alert, anxious appearing
- **HEENT:**
  ○ **Head:** atraumatic
  ○ **Eyes:** eyelids drooping bilaterally, pupils are equal round and reactive to light
  ○ **Ears:** within normal limits
  ○ **Nose:** within normal limits
  ○ **Throat:** within normal limits
- **Neck:** within normal limits
- **Heart:** tachycardic, regular rhythm
- **Lungs:** tachypneic with shallow respirations; lungs clear to auscultation bilaterally
- **Abdominal/GI:** within normal limits
- **Genitourinary:** Deferred
- **Rectal:** Deferred
- **Extremities:** bite wound to the right hand with mild surrounding erythema
- **Back:** within normal limits
- **Neuro:** Awake, alert and oriented to person, place and time. Speech initially clear without dysarthria. Bilateral ptosis present. Pupils 3 mm, equal round and reactive to light. Extraocular movements intact initially. Sensation intact to light touch throughout. Strength 5/5 in all extremities. Deep tendon reflexes 2+ and symmetric. Coordination intact.
  ○ As the scenario progresses without antivenom, the patient develops worsening bulbar weakness, dysarthria, shallow respirations, and declining respiratory effort consistent with progressive neurotoxic envenomation.
- **Skin:** bite wound to the right hand with mild surrounding erythema
- **Lymph:** within normal limits
- **Psych:** within normal limits

Picture of snakebite wound^8^

## OPERATOR MATERIALS

**Diagnosis:** Neurotoxic snakebite followed by anaphylaxis (if antivenom given) or respiratory arrest requiring intubation (if no antivenom given).

**Disposition:** The patient should be admitted for monitoring in an ICU.

## DEBRIEFING AND EVALUATION PEARLS

### Snake Bite with Antivenom-Associated Anaphylaxis

**Pearls:**

- Approximately 45,000 snakebites are reported to U.S poison centers annually, with 7,000–8,000 resulting in venomous envenomation. Most fatal envenomation occurs following rattlesnake bites.^1^
- The most common venomous snakes native to the United States are pit vipers (rattlesnakes, copperheads, and cottonmouths) and coral snakes, while belong to the elapid family.^1^
- Pit viper venom primarily causes local tissue injury, edema, ecchymosis, coagulopathy, and systemic hemotoxic effects.^3^
- Coral snake venom is predominantly neurotoxic, producing cranial nerve dysfunction, ptosis, dysarthria, dysphagia, and respiratory paralysis with minimal local tissue injury.
- Although uncommon, Mojave rattlesnakes and Eastern Diamondback rattlesnakes may also produce clinically significant neurotoxicity despite being pit vipers.^3^
- Approximately 25% of pit viper bites are dry bites, in which no venom is injected. Patients with suspected venomous snakebites require observation because signs of envenomation may be delayed.^1^,^3^
- Progressive swelling, worsening pain, coagulopathy, hypotension, neurologic deficits, or systemic symptoms are consistent with clinically significant envenomation and warrant treatment with antivenom.^1^,^3^
- Antivenom is the primary treatment for clinically significant envenomation. Early administration limits tissue injury and systemic toxicity, although benefit may still occur even when treatment is delayed.^1^,^3^
- Immediate hypersensitivity reactions including anaphylaxis may occur during antivenom administration and should be managed with standard anaphylaxis treatment while balancing the need for continued antivenom therapy. Hypersensitivity reactions are potential complications of antivenom therapy and should not be interpreted as an expected or universal response.^3^,^9^
- Initial laboratory evaluation for suspected venomous snakebite typically includes a CBC, coagulation studies, fibrinogen, electrolytes, renal function, and serial reassessment because laboratory abnormalities may evolve over time.
- **Indian Cobra (*****Naja naja*****) Snake:** The Indian Cobra (*Naja naja*) is a snake native to India and Pakistan.^2^,^10^ The average adult snake measures between 100–150 cm and has a hood typical of all members of their genus.^10^ This hood is not always expanded and is only shown when the snake is agitated or threatened. Its coloration is variable with grey, red, tan, brown and black specimens represented. They are often best known for their binocellate or spectacled pattern on their hood, which appears as two circles connected by a line. However, it can be absent on some members of the species.^2^,^10^ Distinguishing from similar naja species can be difficult and requires close evaluation of their scale pattern and hood markings.^1^,^2^,^10^ Lyophilised Polyvalent Anti Snake Venom Serum, by Haffkine Bio-Pharmaceutical Corporation Ltd., is one of the antivenoms available in India. It is an antivenom that provides coverage against the “The Big Four”: four venomous snakes attributed to cause the most morbidity and mortality in India.^2^ Those snakes are the Indian Cobra (*Naja naja*), Common Krait (*Bungarus caeruleus*), Russell's Viper (*Vipera russelli*) and Saw scaled Viper (*Echis carinatus*). The antivenom is derived from purified equine globulins and dosing is dependent on the severity of the reaction.^1^,^2^,^10^
- In the United States, antivenom availability differs from many countries where snakebites are more common. Antivenom is not routinely stocked in every emergency department and is typically maintained at designated hospitals, zoos, academic centers, or regional repositories due to limited demand, cost, and shelf-life. Regional poison centers can assist with identifying available antivenom and coordinating transfer. The estimated time for antivenom delivery varies depending on geographic location, hospital resources and whether the medication is stocked locally. The primary antivenoms used for native United States snakebites include: Crotalidae Polyvalent Immune Fab (ovine-derived, covers North American pit vipers), Crotalidae Immune F(ab)_2_ (equine-derived, covers North American crotaline snake envenomation), and North American Coral Snake Antivenin (equine-derived, covers coral snake envenomation).^9^,^11^
- **Emergency Medicine ABCs of Snakebites:**
  ○ Airway and breathing: Assess for early airway protection (altered mentation, impending respiratory failure); Confirm bilateral breath sounds and adequate oxygenation/ventilation.
  ○ Circulation: Assess central and peripheral pulses; confirm symmetry. Mark the bite site and document time of marking if swelling or progression is anticipated.
    ▪ Initiate limb immobilization. Pressure immobilization techniques may be considered for selected neurotoxic elapid envenomation (such as cobra bites) according to regional guidelines. However, this approach differs from management of many North American pit viper bites, where routine pressure immobilization is generally not recommended.^12^
- Access and Initial Diagnostics:^3^
  ○ Establish IV access
  ○ Obtain initial laboratory studies to assess for systemic toxicity. Do NOT wait for laboratory results for antivenom administration if there is a high clinical suspicion.
  ○ ***CBC**** – evaluate for thrombocytopenia.*
  ○ ***CMP**** – assess electrolyte abnormalities and hepatic function.*
  ○ ***PT/INR and aPTT**** – evaluate for coagulopathy.*
  ○ ***Fibrinogen**** – assess venom-induced consumptive coagulopathy.*
  ○ ***Creatine phosphokinase (CPK)**** – evaluate for rhabdomyolysis.*
  ○ ***Venous blood gas (VBG)**** – assess for hypercapnia and metabolic derangements.*
- Snakebite First Aid^12^
  ○ Reinforce evidence-based snakebite first aid principles
- Early toxicology consultation recommended to:
  ○ Assist with possible snake identification
  ○ Provide guidance on species-specific envenomation patterns
  ○ Identify pearls and pitfalls in management
  ○ Assist with antivenom selection, acquisition, and administration
- Antivenom Administration^3^,^9^,^11^
  - Antivenom should be reconstituted according to manufacturer package inserts. In general, use gentle swirling or continuous manual inversion. Avoid shaking or vigorous agitation, which can cause foaming, air bubble formation, and potential protein denaturation, complicating accurate administration.
  - ***Adverse Reactions:***
    ○ Place the patient on continuous cardiac and hemodynamic monitoring prior to administration. Antivenom can cause hypersensitivity reactions, including anaphylaxis. Monitor closely during and after infusion; anaphylaxis typically occurs within the first 15 minutes. Routine pretreatment is not generally recommended.^3^,^9^,^11^ Be prepared to recognize and treat anaphylaxis promptly, including administration of epinephrine when indicated.
- Disposition:
  ○ Most patients with clinically significant envenomation require ICU-level care for frequent reassessment, serial examinations, and laboratory monitoring.
  ○ Favor medical admission when possible. Surgical intervention (fasciotomy) is rarely indicated and generally discouraged unless: true compartment syndrome is confirmed by objective compartment pressure measurements AND neurovascular compromise persists despite adequate antivenom administration.^13^
    ▪ Avoid unnecessary fasciotomy: snakebite-associated swelling has historically prompted concern for compartment syndrome. However, routine fasciotomy is generally NOT recommended. Progressive edema and tissue injury should be treated with antivenom and supportive care.^13^

**Other debriefing points:**

- In addition to standardized debriefing tools, consider emphasizing:
  ○ Diagnostic uncertainty and early consultation.
  ○ Team communication around antivenom preparation and monitoring.
  ○ Reassessment and trend recognition (exam findings, labs, progression of swelling).
- If learners did not retain Emergency Medical Services (EMS) at the bedside, ask:
  - *“Were there additional sources of history you could have utilized?”*

If available, consider involving a herpetologist in the simulation or debrief to discuss:

- Their training in snakebite first aid.
- Real-world considerations in species identification and exposure history.

## SIMULATION ASSESSMENT

*Snake Bite with Antivenom-Associated Anaphylaxis*

Learner: _________________________________________

***Assessment Timeline***

This timeline is to help observers assess their learners. It allows observer to make notes on when learners performed various tasks, which can help guide debriefing discussion.

**Critical Actions:**

□ Verbalizes that the Indian Cobra (*Naja naja*) snake produces neurotoxic envenomation and communicates the implications for patient management (need for airway monitoring and early toxicology consultation)
□ Perform the emergency medicine airway, breathing, circulation (“ABC”):
  □ Intubation?
  □ Bilateral breath sounds?
  □ Equal pulses? Mark the site (if needed)? Immobilize affected extremity? Avoidance of tourniquets or incision/suction?
□ IV access and labs ordered: complete metabolic panel (CMP), complete blood count (CBC), fibrinogen, prothrombin time (PT) and partial thromboplastin time (PTT), creatinine phosphokinase (CPK), venous blood gas (VBG)
□ Ask for antivenom (snake handler brings the antivenom with him from the zoo)
□ Perform Snakebite First Aid: Splint the extremity and elevate to the level of the heart
□ Call the Toxicology consult/Snakebite consult
□ Coordinate antivenom administration with pharmacy/nursing team and confirm appropriate preparation and monitoring requirements
□ Connect the patient to the monitor for antivenom administration
□ Monitor for anaphylaxis. Pretreatment is generally not needed.
□ Verbally recognize the development of anaphylaxis (identifies hypotension, urticaria, wheezing, or airway involvement) and promptly administers intramuscular epinephrine
□ Disposition: The patient should be admitted to an appropriate ICU setting for monitoring and management. Specific admitting services and ICU location may vary by institution.

**Summative and formative comments:**

**Milestones assessment:**

## Appendix A Critical Actions Checklist

**Table:**

Critical Actions
□ Identify that the Indian Cobra (*Naja Naja*) snake is neurotoxic
Emergency Medicine ABCs: □ Intubation? □ Bilateral breath sounds? □ Equal pulses? Mark the site (if needed)? Compression of site?
□ IV access and labs ordered : Complete Metabolic Panel (CMP) Complete Blood Count (CBC), Fibrinogen, Prothrombin Time and Partial Thromboplastin Time, (PT/PTTs), Creatine Phosphokinase Test **(**CPK), Venous Blood Gas (VBG, for hypercapnia)
□ Ask for antivenom (snakebite handler brings the antivenom with him from zoo)
□ Perform snakebite First Aid: Splint the extremity and elevate to the level of the heart
□ Call Toxicology consult/Snakebite consult
□ Instruct nursing how to give the antivenom
□ Connect the patient to monitor for antivenom administration
□ Monitor for anaphylaxis (occurs within 15 minutes typically but pretreatment is not generally needed)
□ Recognize anaphylaxis and administer epinephrine
□ Disposition: Critical care consult for ICU admission

## References

[1] Uetz P, Freed P, Aguilar R (2025). The Reptile Database.

[2] Chakma JK, Menon JC, Dhaliwal RS (2020). White paper on venomous snakebite in India. Indian J Med Res.

[3] Kanaan NC, Ray J, Stewart M (2015). Wilderness Medical Society Practice Guidelines for the Treatment of Pitviper Envenomations in the United States and Canada. Wilderness Environ Med.

[4] Matzke CM, DeGennaro R, Howie-Esquivel J (2021). Incorporating TeamSTEPPS training to improve staff collaboration in an academic level I emergency and trauma center. Int Emerg Nurs.

[5] Sharma H, Baviskar A, Patil AD (2024). Enhancing primary healthcare worker training through high-fidelity simulation for snake bite management. J Emerg Trauma Shock.

[6] Wijnen-Meijer M, Brandhuber T, Schneider A, Berberat PO (2022). Implementing Kolb’s Experiential Learning Cycle by linking real experience, case-based discussion and simulation. J Med Educ Curric Dev.

[7] Meguerdichian M, Bajaj K, Ivanhoe R (2022). Impact of the PEARLS Healthcare Debriefing cognitive aid on facilitator cognitive load, workload, and debriefing quality: a pilot study. Adv Simul (Lond).

[8] Fyn Kynd Photography. Wikimedia Commons.

[9] ANAVIP FDA Drug Label.

[10] Wuster W, Wuster W (1998). The cobras of the genus Naja in India. Hamadryad.

[11] CroFab FDA Drug Label.

[12] Modified from: NSW Poisons Information Centre (n.d). Pressure immobilisation. https://www.poisonsinfo.nsw.gov.au/First-Aid/Pressure-Immobilisation.aspx.

[13] Varney SM, Alindogan AA, Stuteville H (2024). Fasciotomy following North American pit viper envenomation in Texas 2004–2021. *Clin Toxicol*(Phila).

